# Supplementary material for: mosGILT antibodies interfere with Plasmodium sporogony in Anopheles gambiae
Source: Nat Commun. 2025 Jan 11;16:592. doi: 10.1038/s41467-025-55902-1 (PMC11724845; doi:10.1038/s41467-025-55902-1)
Supplement: Supplementary file 1 — Supplementary Information [file 41467_2025_55902_MOESM1_ESM.pdf]

Supplemental figures S1-S7

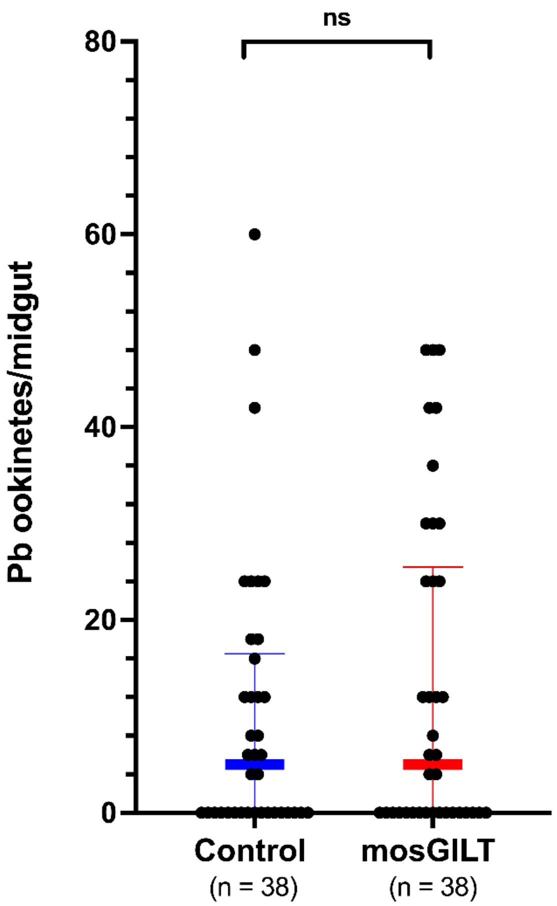

Fig. S1: mosGILT antibodies do not impact the *Plasmodium* ookinete abundance. The intensity of *P. berghei* ookinetes in a blood meal bolus and traversing the midgut epithelium of *A. gambiae* 18 hours after engorging on a *P. berghei*-infected mouse passively immunized with mosGILT (n = 38) or control (n = 38) antisera (*p* = 0.6196). Each dot represents the number of ookinetes in an individual mosquito midgut, and the blue and red horizontal lines indicate the medians for the control and intervention groups, respectively. The thin lines indicate upper and lower quartiles. Two experimental replicates were performed. Two-Tailed Mann-Whitney was used to determine the significance of ookinete abundance (ns not significant).

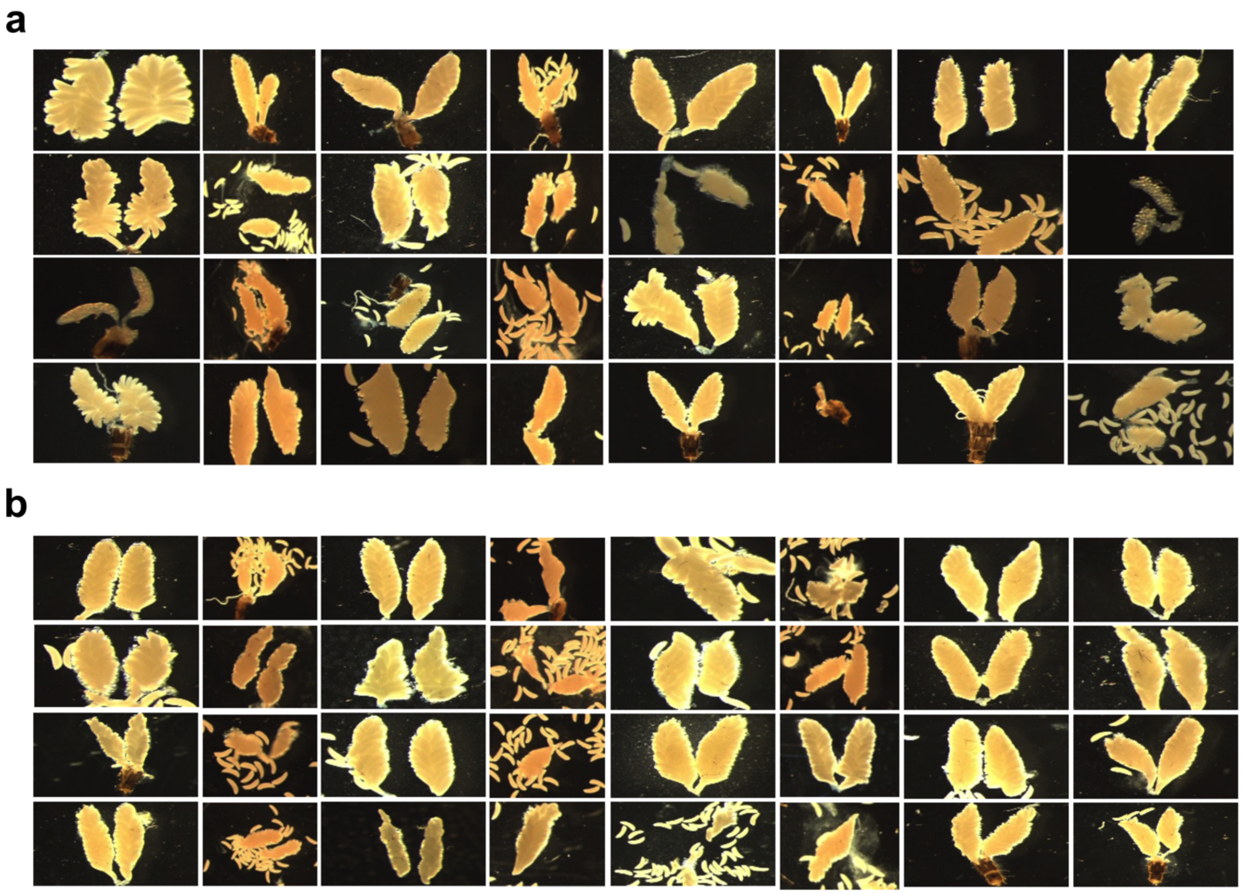

Fig. S2: mosGILT antibodies do not impact ovarian development. Ovaries were dissected from female *A. gambiae* two days after a blood meal on mice passively immunized with control (GST) (**a**) or mosGILT antisera (**b**). Two experimental replicates were performed.

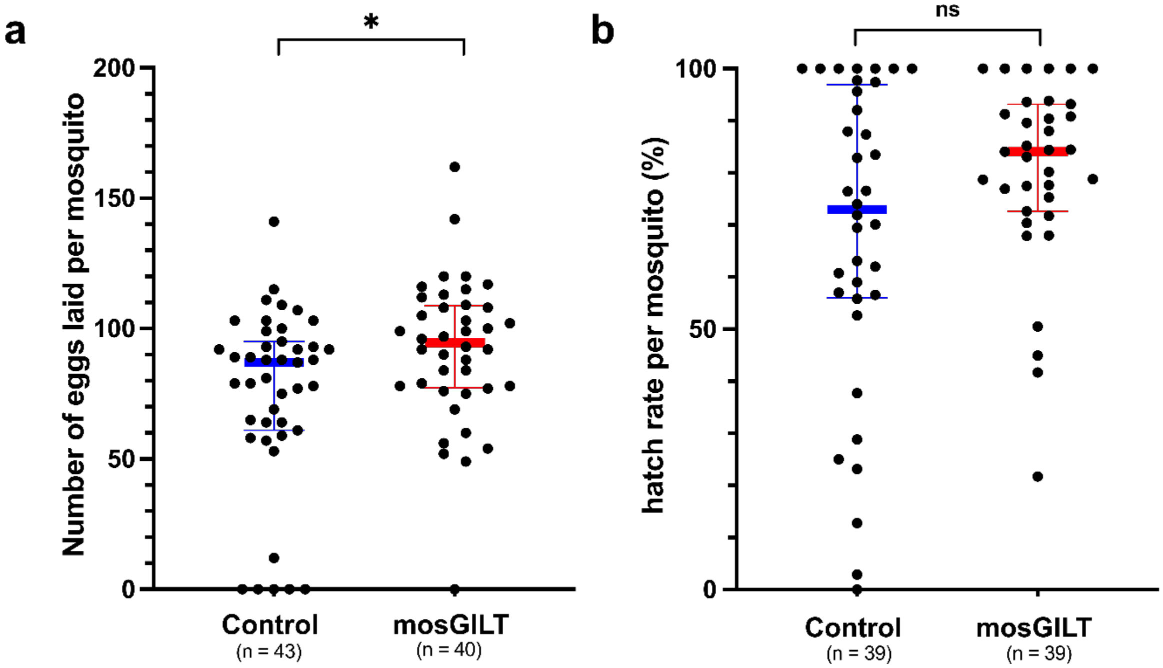

Fig. S3: mosGILT antibodies do not negatively impact female egg laying capacity or hatch rate. *A. gambiae* were fed on mice passively immunized with mosGILT (n = 40; n = 39) or control (n = 43; n = 39) antisera. Five days after infection, the number of eggs laid per mosquito were counted (**a**, p = 0.0236), and the percentage of larvae that hatched from eggs was computed (**b**, $p = 0.1269$). Each dot represents the number of eggs or hatch rate for an individual mosquito, and the blue and red horizontal lines indicate the medians for the control and intervention group, respectively. The thin lines indicate upper and lower quartiles. Two experimental replicates were performed. Two-Tailed Mann-Whitney was used to determine the significance of egg abundance and larval hatch rate (ns not significant, * $p \leq 0.05$).

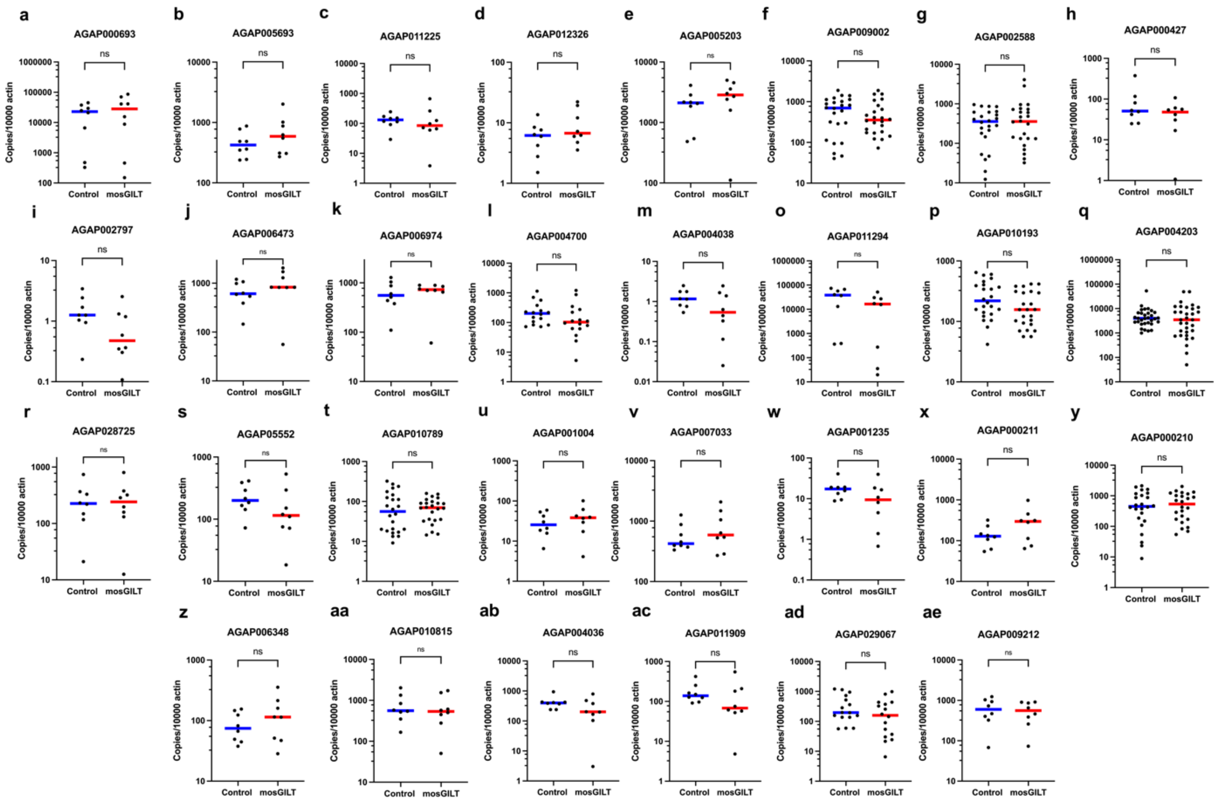

Fig. S4: The most up- and down-regulated genes from mosGILT mutant mosquitoes show no change in expression by RT-qPCR following mosGILT antibody ingestion. a-ae, expression level of gene in midguts 48 hours after engorgement on animals immunized with mosGILT or control antibodies, as determined by RT-qPCR. Each dot represents an individual mosquito midgut, and the blue and red horizontal lines indicate the medians for the control and intervention groups, respectively. Two-Tailed Mann-Whitney was used to determine significance (ns not significant).

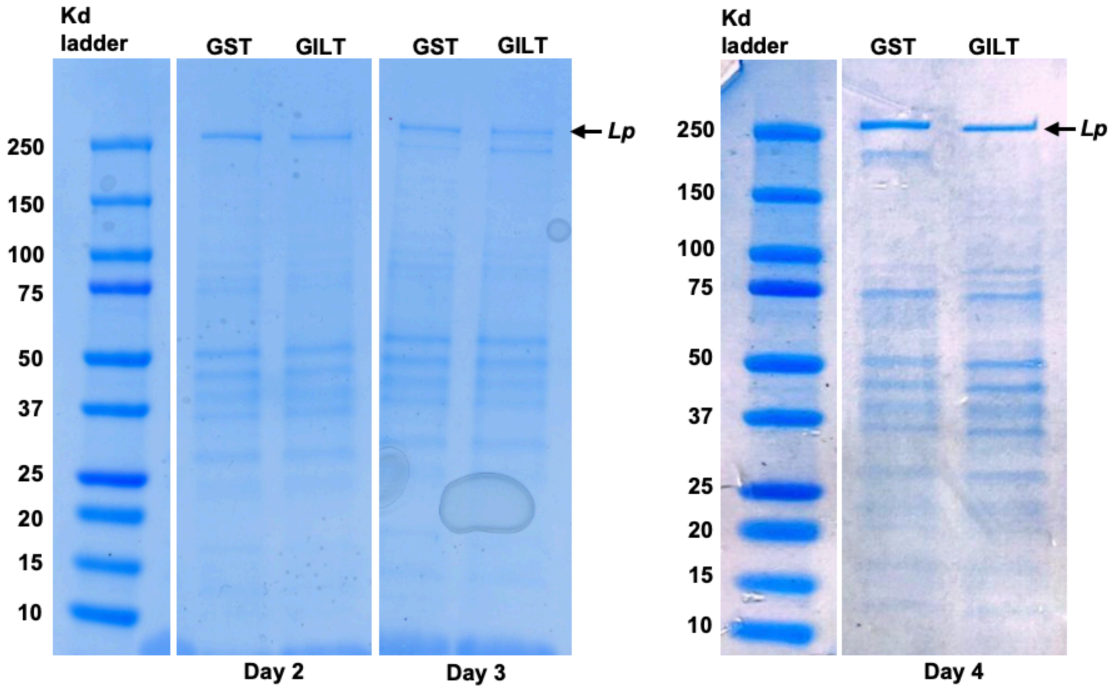

Fig. S5: mosGILT antibodies decrease the *Apolipophorin-I* (*Lp*) protein expression in *A. gambiae*. Mosquitoes were fed on mice that were passively immunized with 400 µl mosGILT or control (GST) antisera. Subsequently, hemolymph droplets from 15 mosquitoes were collected at different time points (2, 3, and 4 days post-blood meal). Proteins in the hemolymph were separated by SDS-PAGE and analyzed by Coomassie staining. Two experimental replicates were performed.

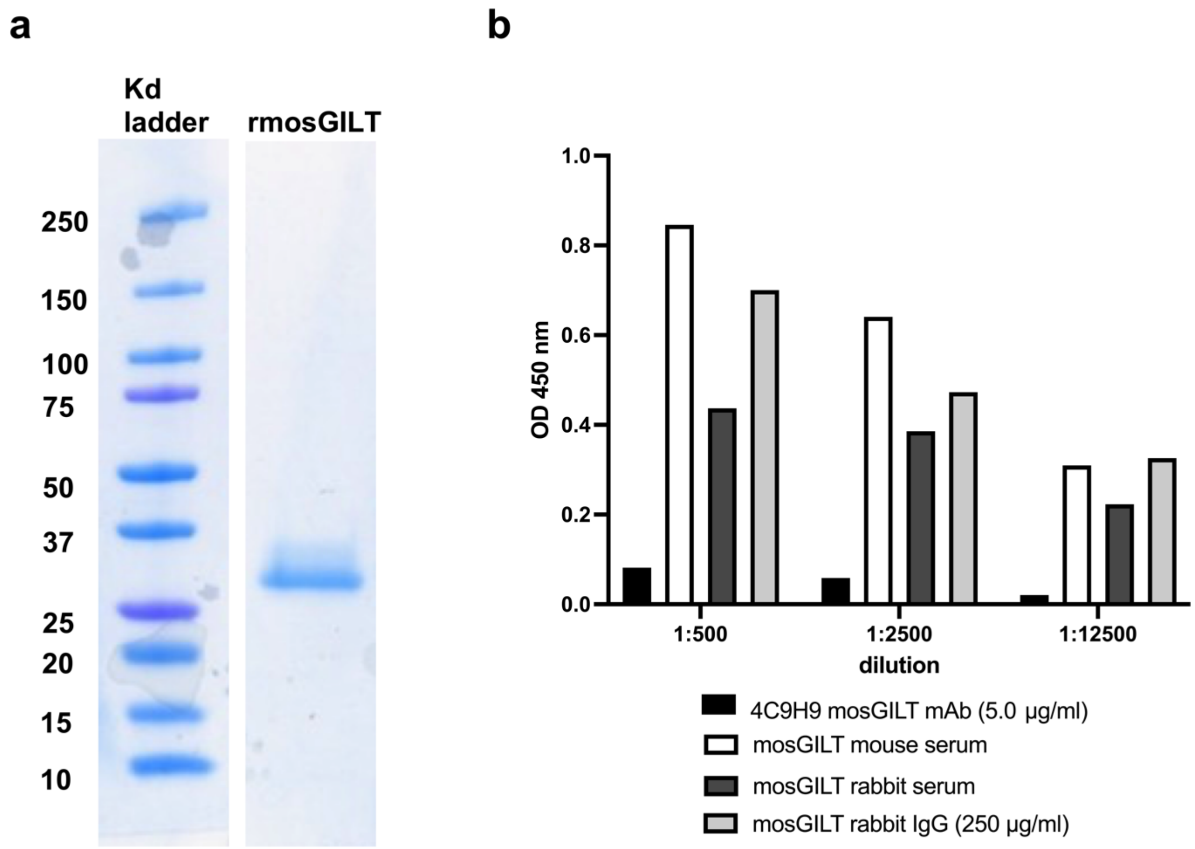

Fig. S6: High purity of recombinant mosGILT antibody and relative activity of generated materials. a, recombinant mosGILT separated by SDS-PAGE and analyzed by Coomassie staining demonstrated high purity of the recombinant protein. **b**, ELISA demonstrates mosGILT-specific binding by the 4C9H9 mosGILT monoclonal antibody, rabbit purified IgG, as well as antibodies in the mosGILT active immunization mouse serum and mosGILT rabbit serum.

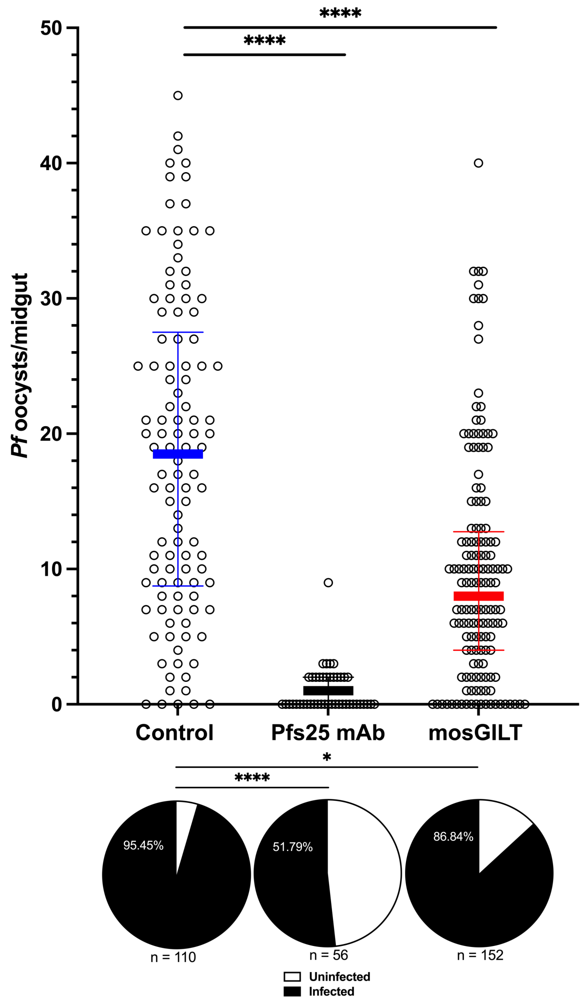

Fig. S7: mosGILT antibodies and Pfs25 monoclonal antibodies (Pfs25 mAb) decrease *P. falciparum* oocyst number and infection prevalence in membrane feeding model. Dot-plot showing the number of *P. falciparum* oocysts per midgut and infection prevalence (pie charts) from mosquitoes that took a *P. falciparum*-infected blood meal with a final concentration of 0.2 mg/ml of control (n = 110) or mosGILT (n = 152) rabbit polyclonal IgG alongside a Pfs25 monoclonal antibody (Pfs25 mAb, n = 56) at 15 µg/ml. Each dot represents the number of oocysts in an individual mosquito midgut. The blue, black, and red horizontal black lines indicate the medians for the control, Pfs25, and mosGILT groups, respectively. The thin lines indicate upper and lower quartiles. Three experimental replicates were performed. Two-Tailed Mann-Whitney was used to determine the significance of oocyst abundance. Fisher's exact test was used to compare infection prevalence values (* $p \leq 0.05$, **** $p \leq 0.0001$). Source data are provided as a Source Data file.

Supplemental tables S1-S2

Table S1. RT-qPCR Primers for *Anopheles gambiae* mosGILT and Actin.

Name	Gene	Forward primer	Reverse primer
mosGILT	AGAP004551	TGGCGAAGGAGCTCAAAAAG	TGCCGTAACACTCGTTTTCG
Actin	AGAP-Actin	GAAGGCTAACCGCGAGAAGATG	CGCCGGAGTCCAGCACGATA

Table S2. Primers for most up- and down-regulated genes from *mosGILT* mutant mosquito RNAseq.

Supplementary table 1			
Name	Gene	Forward primer	Reverse primer
PGRP-LC receptor	AGAP005203	CGGCAAGTCAGATAGCGCTA	ATCGTCACACTCGGTATCGC
Caspar	AGAP006473	GCGATCTATCTGCACCACGA	GACAAGTCCCACCCGTACAG
Toll 9R	AGAP006974	ATCAGAAGCTCGATCCGCTG	CGCCACACTGACCAGTATGT
SPCLIP1	AGAP028725	GCTGATCGACGACTACCTGG	GGCTATTTCCTGCATGCGTG
TEP1	AGAP010815	GCAGCTGAACGCAACAGAAA	ATTTGTCGTCCCGGCTTGAT
PGRPLD	AGAP005552	GCGCTACCTGCAAAATACCG	GTGAAGTTGCCGATCATGCC
SRPN6	AGAP009212	CGCGATGACGACTACGAAGA	GATCGGTTGAATCAGCGCAC
Defensin 1	AGAP011294	AGTGTGCAACGATCGTCTGT	GCTTGGCCCGATAGTTCTCC
GNBP	AGAP002797	CGCTAATGCTCGGCTTCAAC	GGCATGGTAAAATCGACCGC
IGALE	AGAP010193	CGCGAGTACTGCATGGATCT	CTCACGGTTGTTCGACCAGA
Cecropin 1	AGAP000693	CATCTTTGTCGTGCTGGCAG	CTGCCTTGAACACTCGCTTG
LMRN1	AGAP006348	AAGATCACCATGCTGCGTGA	CCAACGTTTTGAGCTTCGCA
APL1C	AGAP007033	GTTGCGACCACCAAACCTTC	TGTACGTTGGACCTCCGTTG
LRRD7	AGAP005693	CGGTTGTTCCAGGGATGTCA	ATTCCGTTCCCGATCGCTAC
Toll 7	AGAP012326	GGGCTGAAGGTGCTGAATCT	TGAACTCGAGGTTGAGGTGC
Toll 1	AGAP001004	GGAACAGCTGACCGATCACA	TGTTGTTGCGCGTAAGGTTG
Hemo peroxidase 7	AGAP004036	ACGGTGAATACTTGCCCAGG	TGGTGTGCGGACTGTAGAAC
Hemo peroxidase 8	AGAP004038	CTGTGTCCTGCGAGTGATGT	CAGTTCAAACGGCAGTGAGC
MEGF10	AGAP010789	TGCCAGGTAGCGAATTCGTT	CCACCCAGCTGTACCGATTT
UNC93B1	AGAP002588	GATACGACACGGTCCCATCC	ATCACGTGAGGCTTCGGATC
Fibrinogen C terminal	AGAP011225	TTCGCTCGTGTAAGGCAGAG	GTACTCGGTCCAGTTGCGAT
Serine protease	AGAP029067	ATATGACAACCTGCCGGCAA	AGTAGCGCTTTTGTCCGTCA
Serine protease	AGAP011909	CCCTACACAGCCCTACTCCT	ACACCGGCATTGAACAGGAT
Phosphatidylserine decarboxylase	AGAP001235	GGCCGATCAAAGAGTTCCCA	CACACGGTGACACAAAGCTG
Trypsin serine like protease	AGAP004700	GTTCCGCGGTGTTCATAAGC	CGGCGACCACCTTCGTAATA
Lp receptor	AGAP001826	GTCGATCTGCACAATGACGC	GAACTTCTTGGCCCCTTCGA
Vg	AGAP004203	CCGACTACGACCAGGACTTC	CTTCCGGCGTAGTAGACGAA
Vg receptor	AGAP000427	ACCAAGAGCATCCAGTCGTG	CGGCTGTATGCTCGAGTGAT
Hormone receptor 3	AGAP009002	CAGCGATCAGTTGCATCACG	CCGAGATGTCGTAGCCCATC
EcR	AGAP029539	TGCCAACAACCGATCGTACA	AGAAGATGACGATCGCGGTC
TAG lipase 2	AGAP000211	AAGATGTCGTTCGAGCTGCA	TTCGTCCGATACTCCCAGGT
TAG lipase 1	AGAP000210	TACTTCATCACGCACGGCTT	TTGTACGGTGGATTGGAGCC

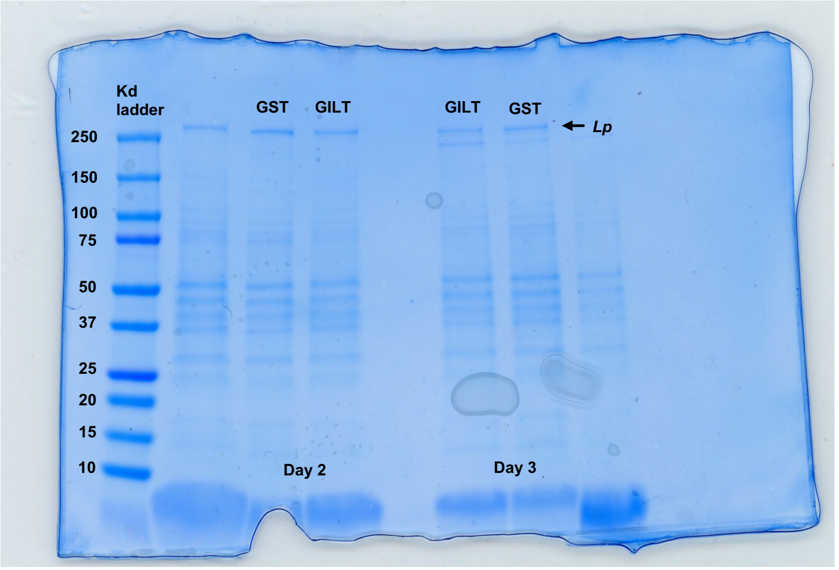
Kd
ladder
GST
GILT
GILT
GST
Lp
250
150
100
75
50
37
25
20
15
10
Day 2
Day 3

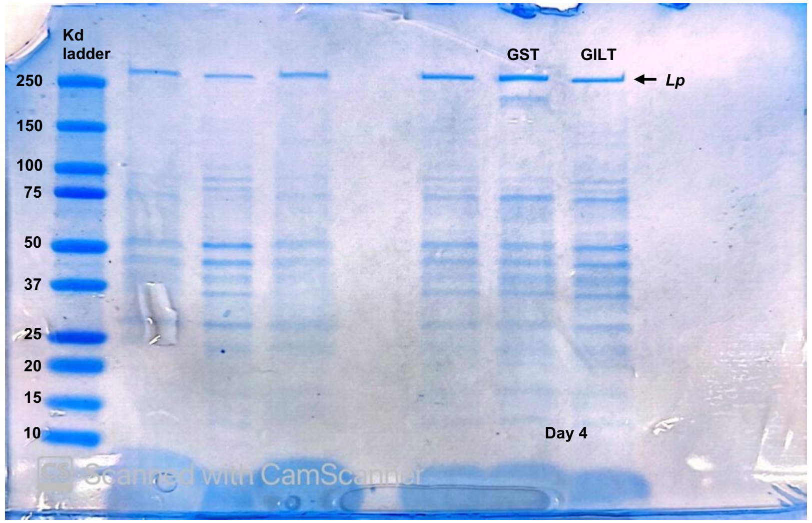
Kd ladder
250
150
100
75
50
37
25
20
15
10
GST
GILT
Lp
Day 4
Scanned with CamScanner

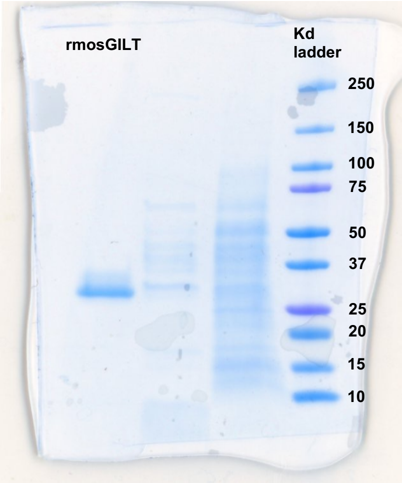
rmosGILT
Kd
ladder
250
150
100
75
50
37
25
20
15
10
