# Supplementary material for: mosGILT antibodies interfere with Plasmodium sporogony in Anopheles gambiae
Source: Nat Commun. 2025 Jan 11;16:592. doi: 10.1038/s41467-025-55902-1 (PMC11724845; doi:10.1038/s41467-025-55902-1)
Supplement: Supplementary file 2 — Reporting summary [file 41467_2025_55902_MOESM2_ESM.pdf]

nature portfolio

Corresponding author(s): Erol Fikrig
Yu-Min Chuang

Last updated by author(s): Dec 13, 2024

Reporting Summary

Nature Portfolio wishes to improve the reproducibility of the work that we publish. This form provides structure for consistency and transparency in reporting. For further information on Nature Portfolio policies, see our Editorial Policies and the Editorial Policy Checklist.

Statistics

For all statistical analyses, confirm that the following items are present in the figure legend, table legend, main text, or Methods section.

n/a	Confirmed	
☐	☒	The exact sample size (*n*) for each experimental group/condition, given as a discrete number and unit of measurement
☐	☒	A statement on whether measurements were taken from distinct samples or whether the same sample was measured repeatedly
☐	☒	The statistical test(s) used AND whether they are one- or two-sided<br>*Only common tests should be described solely by name; describe more complex techniques in the Methods section.*
☒	☐	A description of all covariates tested
☒	☐	A description of any assumptions or corrections, such as tests of normality and adjustment for multiple comparisons
☐	☒	A full description of the statistical parameters including central tendency (e.g. means) or other basic estimates (e.g. regression coefficient) AND variation (e.g. standard deviation) or associated estimates of uncertainty (e.g. confidence intervals)
☐	☒	For null hypothesis testing, the test statistic (e.g. *F*, *t*, *r*) with confidence intervals, effect sizes, degrees of freedom and *P* value noted<br>*Give P values as exact values whenever suitable.*
☒	☐	For Bayesian analysis, information on the choice of priors and Markov chain Monte Carlo settings
☒	☐	For hierarchical and complex designs, identification of the appropriate level for tests and full reporting of outcomes
☒	☐	Estimates of effect sizes (e.g. Cohen's *d*, Pearson's *r*), indicating how they were calculated

Our web collection on statistics for biologists contains articles on many of the points above.

Software and code

Policy information about availability of computer code

Data collection	Data was collected in GraphPad Prism Version 10.1.1
Data analysis	Data was analyzed in GraphPad Prism Version 10.1.1 as well as FIJI.

For manuscripts utilizing custom algorithms or software that are central to the research but not yet described in published literature, software must be made available to editors and reviewers. We strongly encourage code deposition in a community repository (e.g. GitHub). See the Nature Portfolio guidelines for submitting code & software for further information.

Data

Policy information about availability of data

All manuscripts must include a data availability statement. This statement should provide the following information, where applicable:

- Accession codes, unique identifiers, or web links for publicly available datasets
- A description of any restrictions on data availability
- For clinical datasets or third party data, please ensure that the statement adheres to our policy

The authors declare that all other data supporting the findings of this study are available within the article and its Supplementary Information file.

April 2023

Research involving human participants, their data, or biological material

Policy information about studies with human participants or human data. See also policy information about sex, gender (identity/presentation), and sexual orientation and race, ethnicity and racism.

Reporting on sex and gender	Biological material was from a de-identified pool.
Reporting on race, ethnicity, or other socially relevant groupings	Biological material was from a de-identified pool.
Population characteristics	de-identified pre-screened donor population.
Recruitment	Human blood for P. falciparum cultures and mosquito infections was collected from a pool of de-identified pre-screened donors.
Ethics oversight	Institutional Review Board at Johns Hopkins University (protocol NA00019050).

Note that full information on the approval of the study protocol must also be provided in the manuscript.

Field-specific reporting

Please select the one below that is the best fit for your research. If you are not sure, read the appropriate sections before making your selection.

☒ Life sciences ☐ Behavioural & social sciences ☐ Ecological, evolutionary & environmental sciences

For a reference copy of the document with all sections, see nature.com/documents/nr-reporting-summary-flat.pdf

Life sciences study design

All studies must disclose on these points even when the disclosure is negative.

Sample size	Mosquito, egg, larvae, and parasite numbers were counted by eye or using a EVOS fluorescent microscope. No sample-size calculation was performed. Mosquito sample sizes were based on mosquito and reagent availability as well as sample sizes used in prior transmission blocking experiments (Kapulu et al., Sci Rep 5015) (Dinglasan et al., PNAS 2007) (Mlambo et al., Infect Immun 2008).
Data exclusions	No data were excluded from the analyses.
Replication	All experiments were replicated at least twice.
Randomization	Mosquitoes were randomly assigned to control or intervention groups.
Blinding	Investigators were blinded to treatment condition during data collection and analysis of the in vivo transmission-blocking experiments.

Reporting for specific materials, systems and methods

We require information from authors about some types of materials, experimental systems and methods used in many studies. Here, indicate whether each material, system or method listed is relevant to your study. If you are not sure if a list item applies to your research, read the appropriate section before selecting a response.

Materials & experimental systems

n/a	Involved in the study	
☐	☒	Antibodies
☒	☐	Eukaryotic cell lines
☒	☐	Palaeontology and archaeology
☐	☒	Animals and other organisms
☒	☐	Clinical data
☒	☐	Dual use research of concern
☒	☐	Plants

Methods

n/a	Involved in the study	
☒	☐	ChIP-seq
☒	☐	Flow cytometry
☒	☐	MRI-based neuroimaging

Antibodies

Antibodies used	Invitrogen Goat anti-Rabbit IgG (H+L) Secondary Antibody, HRP (Product # 31460)

	Invitrogen Goat anti-Mouse IgG1 Secondary Antibody, Alexa Fluor™ 647 (Product # A-21240)<br><br>GenScript generated monoclonal antibody 4C9H9 against recombinant mosGILT protein.<br><br>The following reagent was obtained through BEI Resources, NIAID, NIH: Monoclonal Anti-Plasmodium falciparum 25-kDa Gamete Surface Protein (Pfs25), Clone 4B7 (produced in vitro), MRA-28, contributed by Louis H. Miller and Allan Saul.”
Validation	Invitrogen Goat anti-Rabbit IgG (H+L) Secondary Antibody, HRP (Product # 31460) -<br><br>Reference from manufacturer:<br><br>"Product # 31460 has been successfully used in Western blot, IHC and IP applications"<br><br>Dai, S., Li, H., Li, L., Song, Z., Zhang, X., Wang, P., & Wang, H. (2024). Prognostic significance of elevated expression levels of protein phosphatase 1 regulatory subunit 3G in thyroid carcinoma. Oncology letters, 29(2), 82. https://doi.org/10.3892/ol.2024.14828<br><br>Invitrogen Goat anti-Mouse IgG1 Secondary Antibody, Alexa Fluor™ 647 (Product # A-21240) -<br><br>"To minimize cross-reactivity, these goat anti-mouse IgG1 whole secondary antibodies have been affinity purified and crossadsorbed against mouse mouse IgM, mouse IgA, pooled human sera, purified human paraproteins, and mouse isotypes IgG2a, IgG2b, and IgG3 prior to conjugation. Cross-adsorption or pre-adsorption is a purification step to increase specificity of the antibody resulting in higher sensitivity and less background staining. The secondary antibody solution is passed through a column matrix containing immobilized serum proteins from potentially cross-reactive species. Only the nonspecific-binding secondary antibodies are captured in the column, and the highly specific secondaries flow through. The benefits of this extra step are apparent in multiplexing/ multicolor-staining experiments (e.g., flow cytometry) where there is potential cross-reactivity with other primary antibodies or in tissue/cell fluorescent staining experiments where there are may be the presence of endogenous immunoglobulins."<br><br>Reference from manufacturer:<br><br>Jones, R. A., Ramirez-Bencomo, F., Whiting, G., Fang, M., Lavender, H., Kurzyp, K., Thistlethwaite, A., Stejskal, L., Rashmi, S., Jerse, A. E., Cehovin, A., Derrick, J. P., & Tang, C. M. (2024). Tackling immunosuppression by Neisseria gonorrhoeae to facilitate vaccine design. PLoS pathogens, 20(11), e1012688. https://doi.org/10.1371/journal.ppat.1012688<br><br>Monoclonal Anti-Plasmodium falciparum 25-kDa Gamete Surface Protein (Pfs25), Clone 4B7 (produced in vitro) -<br><br>"Monoclonal antibody 4B7 is reported to function in immunoblot, SDS-PAGE and transmission blocking analysis."

Animals and other research organisms

Policy information about studies involving animals; ARRIVE guidelines recommended for reporting animal research, and Sex and Gender in Research

Laboratory animals	Adult Female Anopheles gambiae (4arr strain, MRA-121, MR4, ATCC; Keele strain)<br>8-week-old female Swiss Webster mice were purchased from Charles River Laboratories (Wilmington, MA).<br>8-week-old female SC57BL/6 mice were purchased from Charles River Laboratories (Wilmington, MA).
Wild animals	Study did not involve wild animals.
Reporting on sex	N/A
Field-collected samples	Study did not involve samples collected from the field.
Ethics oversight	The animal experimental protocol was approved by the Institutional Animal Care and Use Committee of Yale University (protocol permit no. 2023–07941).

Note that full information on the approval of the study protocol must also be provided in the manuscript.

Plants

Seed stocks	N/A
Novel plant genotypes	N/A
Authentication	N/A
