# Supplementary material for: mosGILT antibodies interfere with Plasmodium sporogony in Anopheles gambiae
Source: Nat Commun. 2025 Jan 11;16:592. doi: 10.1038/s41467-025-55902-1 (PMC11724845; doi:10.1038/s41467-025-55902-1)
Supplement: Supplementary file 4 — Source Data [file 41467_2025_55902_MOESM4_ESM.zip › Source Data 2/Complete blot for Fig. 1a. .zip!/Complete blot for Fig. 1a. .pdf]

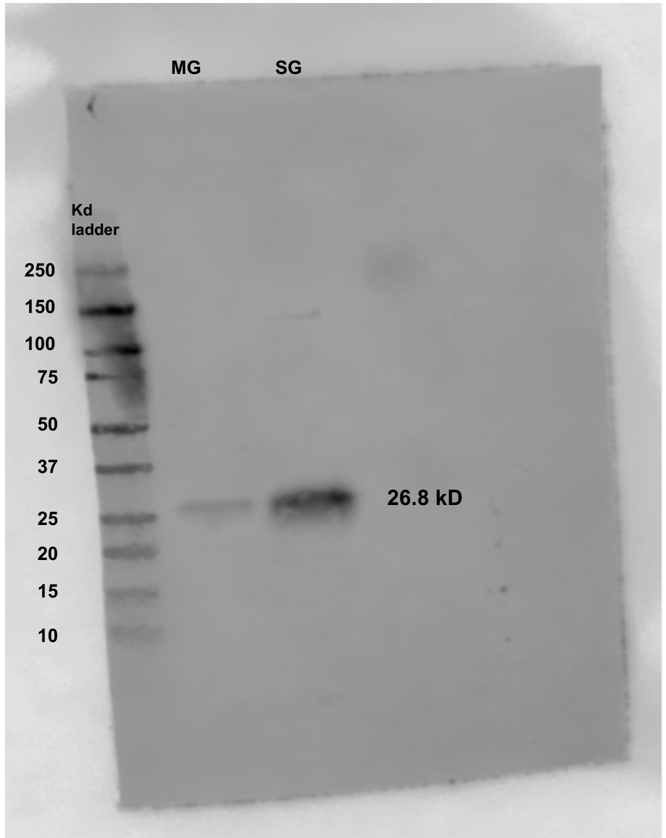
MG
SG
Kd ladder
250
150
100
75
50
37
25
20
15
10
26.8 kD
