# Supplementary material for: mosGILT antibodies interfere with Plasmodium sporogony in Anopheles gambiae
Source: Nat Commun. 2025 Jan 11;16:592. doi: 10.1038/s41467-025-55902-1 (PMC11724845; doi:10.1038/s41467-025-55902-1)
Supplement: Supplementary file 4 — Source Data [file 41467_2025_55902_MOESM4_ESM.zip › Source Data 2/Statistical analysis output for Fig. 2, 4, 5c, 6c, & 7c.zip!/Statistical analysis output for Fig. 2, 4, 5c, 6c, & 7c..pdf]

**Fig. 2a (*Pf oocysts*)**	**Control**	**mosGILT**
n =	51	66
range	0,1-27	0,1-32
Prevalence (%)	92.16	65.15
Fisher’s exact test p-value		0.0007(***)
median (with zeros)	3.0	1.1
Mann-Whitney test p-value		0.0009(***)
% decreased oocyst #		63.3
median (without zeros)	4.0	4.0
Mann-Whitney test p-value		0.2678 (ns)
% decreased oocyst #		N/A

**Fig. 2b (*Pf oocysts*)**	**Control**	**mosGILT**
n =	78	89
range	0,1-150	0,1-115
Prevalence (%)	94.87	83.15
Fisher’s exact test p-value		0.0260 (*)
median (with zeros)	24.5	8.0
Mann-Whitney test p-value		0.0048 (**)
% decreased oocyst #		67.3
median (without zeros)	29.5	13.5
Mann-Whitney test p-value		0.0729 (ns)
% decreased oocyst #		N/A

**Fig. 2c (*Pb oocysts*)**	**Control**	**mosGILT**
n =	152	179
range	0,1-35	0,1-5
Prevalence (%)	30.92	10.60
Fisher’s exact test p-value		<0.0001 (****)
median (with zeros)	0.0	0.0
Mann-Whitney test p-value		<0.0001 (****)
% decreased oocyst #		N/A
median (without zeros)	2.0	1.0
Mann-Whitney test p-value		0.0157 (*)
% decreased oocyst #		50

**Fig. 4a (*Pb oocysts*)**	**Control**	**mosGILT**
n =	75	54
range	0,1-38	0,1-10
Prevalence (%)	53.33	12.96
Fisher’s exact test p-value		<0.0001 (****)
median (with zeros)	1.0	0.0
Mann-Whitney test p-value		<0.0001 (****)
% decreased oocyst #		100.0
median (without zeros)	3.0	2.0
Mann-Whitney test p-value		0.5593 (ns)
% decreased oocyst #		N/A

**Fig. 4b (*Pb oocysts*)**	**Control**	**mosGILT**
n =	75	76
range	0,1-161	0,1-170
Prevalence (%)	74.67	55.26
Fisher’s exact test p-value		0.0168 (*)
median (with zeros)	5.0	1.0
Mann-Whitney test p-value		0.0025 (**)
% decreased oocyst #		80.0
median (without zeros)	22.5	11.0
Mann-Whitney test p-value		0.0888 (ns)
% decreased oocyst #		N/A

**Fig. 4c (*Pb oocysts*)**	**Control**	**mosGILT**
n =	41	43
range	0,1-8	0,1-6
Prevalence (%)	65.85	16.28
Fisher’s exact test p-value		<0.0001 (****)
median (with zeros)	1.0	0.0
Mann-Whitney test p-value		<0.0001 (****)
% decreased oocyst #		100.0
median (without zeros)	2.0	1.0
Mann-Whitney test p-value		0.1215 (ns)
% decreased oocyst #		N/A

**Fig. 5c (*Pb oocysts*)**	**Control**	**mosGILT**
n =	72	118
range	0,1-270	0,1-147
Prevalence (%)	79.17	37.29
Fisher’s exact test p-value		<0.0001 (****)
median (with zeros)	7.5	0.0
Mann-Whitney test p-value		<0.0001 (****)
% decreased oocyst #		100.0
median (without zeros)	19.0	7.0
Mann-Whitney test p-value		0.0046 (**)
% decreased oocyst #		63.16

**Fig. 6c (*Pb oocysts*)**	**Control**	**mosGILT**
n =	51	76
range	0,1-61	0,1-56
Prevalence (%)	74.51	51.23
Fisher’s exact test p-value		0.0099 (**)
median (with zeros)	5.0	1.0
Mann-Whitney test p-value		0.0001 (***)
% decreased oocyst #		80.0
median (without zeros)	8.5	3.0
Mann-Whitney test p-value		0.0026 (**)
% decreased oocyst #		64.7

**Fig. 7b (*Pb oocysts*)**	**Control**	**mosGILT**
n =	35	29
range	0,1-200	0,1-25
Prevalence (%)	85.71	72.41
Fisher’s exact test p-value		0.2236 (ns)
median (with zeros)	9.0	3.0
Mann-Whitney test p-value		0.0060 (**)
% decreased oocyst #		66.7
median (without zeros)	11.0	3.0
Mann-Whitney test p-value		0.01 (**)
% decreased oocyst #		72.7
